# Molecular Mechanisms of Blood–Membrane Interactions in Hemodialysis: Advances in Anti-Inflammatory Surface Modifications

**DOI:** 10.3390/membranes16100332

**Published:** 2026-10-03

**Authors:** Jumiao Wang, Xiaoqi Jiang, Xiuyan Liu, Peiqiang Pan

**Affiliations:** 1 Department of Cardiac Surgery, Qingdao Central Hospital University of Health and Rehabilitation Sciences, No. 127 Siliu South Road, Shibei District, Qingdao 266042, China; 18266231865@163.com; 2 Blood Purification Center, Qingdao Central Hospital University of Health and Rehabilitation Sciences, No. 127 Siliu South Road, Shibei District, Qingdao 266042, China; 17771060439@163.com (X.J.); 13792860206@163.com (X.L.); 3 Department of Nephrology, Qingdao Central Hospital University of Health and Rehabilitation Sciences, No. 127 Siliu South Road, Shibei District, Qingdao 266042, China

**Keywords:** hemodialysis membrane, biocompatibility, blood–membrane interaction, molecular mechanisms, anti-inflammatory modification, complement activation

## Abstract

The development of novel hemodialysis membranes aims not only to enhance dialysis efficiency but also to improve hemocompatibility, an important determinant of treatment safety and tolerability. This narrative review provides a comprehensive overview of the molecular mechanisms underlying blood–membrane interactions during hemodialysis, focusing on key processes such as protein adsorption, complement activation, platelet and leukocyte responses, oxidative stress, and inflammatory mediator release. These interconnected processes can contribute to acute blood–material incompatibility and thrombo-inflammatory responses during dialysis, although their contribution to long-term clinical outcomes remains multifactorial and incompletely defined. Furthermore, the review critically examines current surface-modification strategies, including hydrophilic and antifouling modifications, antithrombotic and complement-directed interfaces, and antioxidant functionalization. Particular emphasis is placed on distinguishing mechanistic and surrogate findings from clinically meaningful evidence and on considering hemocompatibility together with membrane transport performance. By integrating molecular mechanisms, modification strategies, and translational evidence, this review aims to provide a framework for the development and evaluation of safer and more effective hemodialysis membranes, while avoiding overinterpretation of isolated biomarker changes as established clinical benefit.

## 1. Introduction

Hemodialysis (HD) sustains life in kidney failure by combining diffusion and ultrafiltration across thousands of hollow fibers [1,2]. The membrane must provide adequate removal of uremic solutes and water while limiting albumin loss, hemolysis, thrombosis, and immune activation [3,4]. In this setting, hemocompatibility is not an intrinsic label attached to a polymer [4,5]. It is the response of blood to a complete dialyzer—membrane chemistry and geometry, additives, housing, sterilization, priming, anticoagulation, blood and dialysate flow, and the clinical state of the patient all matter [1,4,5].

The first seconds of blood contact establish a dynamic adsorbed-protein layer [3,4]. Complement and contact pathways then interact with platelets and leukocytes, generating mediators that may be detected during a treatment [5,6,7]. Repetition of even modest activation has been proposed as one component of the chronic inflammatory phenotype of maintenance HD [3,7,8]. That proposition is biologically credible, but it should not be overstated. Persistent inflammation in kidney failure also reflects retained uremic solutes, oxidative stress, volume overload, infection, vascular access, comorbidity, loss of kidney endocrine functions, and exposure to the broader extracorporeal circuit [5,8,9]. A change in C3a, cytokines, or oxidative biomarkers during one session therefore does not, by itself, demonstrate that a membrane causes long-term cardiovascular disease.

Replacement of unsubstituted cellulose by modified cellulose and synthetic polymers markedly reduced some acute bioincompatibility reactions [2,4,8]. Contemporary polysulfone (PSf), polyethersulfone (PES), polyacrylonitrile-derived, polymethylmethacrylate, and cellulose triacetate membranes are nevertheless heterogeneous [1,2,4]. Products sharing a polymer name can differ in hydrophilic additives, surface charge, roughness, pore architecture, fiber geometry, sterilization, and extractables [1,3,10]. Consequently, broad statements that one polymer class is universally “anti-inflammatory” are less informative than product-level characterization under clinically relevant conditions.

Surface modification is attractive because it aims to alter the blood-facing interface without sacrificing bulk transport properties [3,11]. Yet the evidence spans very different stages—from molecular docking and static protein assays to dynamic whole-blood loops, small crossover studies, and a few clinical trials [3,11,12]. This narrative review therefore aims to integrate the linked molecular and cellular events initiated at the blood–membrane interface with the major surface-modification strategies currently under investigation, while distinguishing evidence derived from laboratory models, complete dialyzers, and clinical studies. In contrast to reviews that primarily catalogue membrane materials or modification techniques, particular emphasis is placed here on the strength and translational maturity of the available evidence, the distinction between mechanistic or surrogate findings and clinically meaningful outcomes, and the need to evaluate hemocompatibility together with membrane transport performance.

## 2. Molecular Mechanisms at the Blood–Membrane Interface

### 2.1. Protein Adsorption and Formation of the Secondary Membrane

Plasma proteins reach the membrane within seconds through convection and diffusion [4,13]. The resulting protein corona is dynamic: abundant proteins can be displaced by proteins with greater surface affinity, and adsorption may expose epitopes that were less accessible in solution [13,14,15]. Amount alone is therefore an incomplete descriptor; composition, conformation, orientation, exchange kinetics, and retention under shear all influence the biological identity of the interface (Figure 1) [4,14,16].

Proteomic studies of used dialyzers demonstrate selective rather than purely concentration-driven adsorption [17,18,19]. Albumin, apolipoproteins, immunoglobulins, fibrinogen, complement proteins, and coagulation-related proteins can be recovered in different proportions from different membranes [17,18,20]. These findings support a mechanistic role for the adsorbed layer, but they do not justify a simple dichotomy in which albumin is always protective and fibrinogen is always harmful [15,21]. Adsorbed albumin can undergo conformational change, while fibrinogen-mediated platelet responses depend on the accessibility of receptor-binding domains [15,21,22]. Moreover, protein recovery after dialysis is shaped by extraction methods and does not directly measure the structure of the layer during treatment [16,17,19].

Hydration, charge, and nanoscale topography influence adsorption together rather than independently [14,22,23]. A stable hydration layer can reduce direct protein–polymer contact, whereas hydrophobic domains and charged sites may favor stronger binding or altered conformation [14,24,25]. Roughness can increase available area, but its effect depends on the scale of the features relative to proteins and cells [22,23]. For that reason, contact angle, zeta potential, roughness, or surface chemistry measured on a fresh flat laboratory film cannot be assumed to represent the blood-facing surface of a finished hollow-fiber dialyzer after manufacturing, sterilization, storage, and clinical preparation.

The adsorbed layer is also a functional “secondary membrane” [3,26]. It may change sieving and hydraulic resistance during a session, mask the original surface, and provide ligands for platelets, leukocytes, and complement (Figure 2) [26]. Thus, a successful antifouling modification must be evaluated for both biological activation and transport stability; reducing one selected protein in a static assay is insufficient.

### 2.2. Complement and Contact-System Activation

Complement activation during HD is often described as an alternative-pathway phenomenon, but the current model is broader [6,27,28]. C3 fragments and properdin can support alternative-pathway amplification, while adsorption of ficolin-2 and related recognition molecules implicates the lectin pathway; the classical pathway may contribute in particular contexts [6,27,28,29]. These routes converge at C3 and C5, generating C3a, C5a, opsonic C3 fragments, and soluble or surface-associated terminal complement complexes (TCCs) (Figure 3) [6,30].

C3a and C5a recruit and prime leukocytes, while deposited C3 fragments engage complement receptors [6,30]. C5a can promote neutrophil tissue-factor expression and platelet–neutrophil interactions, providing a mechanistic bridge between inflammation and thrombosis [6,31]. These interactions illustrate that complement activation, contact/coagulation pathways, platelets, and leukocytes form an interconnected thrombo-inflammatory network rather than a simple linear cascade (Figure 3). Early transient leukopenia observed with strongly complement-activating membranes is consistent with pulmonary sequestration and cell adhesion, although contemporary responses are usually smaller than those reported with older cellulose membranes [4,32].

Complement activation is closely interconnected with the adsorbed-protein layer, coagulation-related processes, and downstream cellular responses [6,26,30]. In ex vivo comparisons, surface charge and the evolution of albumin sieving—a surrogate for secondary-membrane formation—have been associated with C3a, C5a, and sC5b-9 generation [26]. Such associations are informative but remain product- and model-specific. Anticoagulant type, blood collection, tubing, air contact, and sampling time can activate or suppress the same pathways and must be controlled before differences are attributed to the membrane.

### 2.3. Platelets, Leukocytes, Oxidative Responses, and Cytokines

Adsorbed fibrinogen and other adhesive proteins can engage platelet integrins and support adhesion, shape change, degranulation, and procoagulant activity [10,15]. Studies of PSf membranes have linked fibrinogen adsorption to activation of platelets and neutrophils through their respective integrin receptors [10,15]. Platelet-derived mediators and P-selectin then facilitate platelet–leukocyte aggregates, which integrate coagulation with innate immune signaling (Figure 3) [33,34]. These platelet–leukocyte interactions also provide a cellular interface through which complement- and coagulation-related signals can reinforce one another, further supporting a network rather than a strictly sequential model of thrombo-inflammatory activation (Figure 3).

Neutrophils respond to complement fragments, platelet signals, and the adsorbed surface by increasing CD11b activation, degranulation, and reactive oxygen species production (Figure 3) [6,10,34]. A hydrophilic PSf membrane showed reduced platelet–neutrophil complex formation and subsequent neutrophil activation in comparative experiments [34]. Circulating markers consistent with neutrophil extracellular trap formation may increase during hemodialysis and have been associated with endothelial dysfunction and vascular-access thrombosis [35,36,37]. However, stimulated neutrophil extracellular trap-forming capacity may be impaired, and uremia, infection, vascular access, and dialysis modality complicate attribution of these findings to the membrane itself [35,36,37,38].

Hemodialysis and the uremic milieu are associated with dysregulation of nuclear factor kappa B- and mitogen-activated protein kinase-related signaling in circulating immune cells, with corresponding changes in inflammatory mediator release [30,39,40]. Interpretation of circulating cytokines is particularly difficult: a cytokine may be produced, adsorbed to the membrane, redistributed, diluted, or removed during the same session [41,42,43]. Differences in interleukin-6 (IL-6) or tumor necrosis factor (TNF) levels measured before and after one treatment therefore indicate net concentration change, not necessarily cellular production at the membrane [41,44,45]. Cellular activation markers, transcript or phosphoprotein responses, and mass-balance measurements can provide stronger mechanistic resolution than plasma concentration alone [16,40,43,46].

### 2.4. Why the Complete Dialyzer Matters

Membrane chemistry is only one determinant of the blood response [3,4]. Hydrophilic additives, their distribution at the blood-facing surface, pore size and asymmetry, fiber inner diameter, wall thickness, surface roughness, zeta potential, potting compounds, housing, and sterilization can all alter exposure [3,47,48]. Polyvinylpyrrolidone (PVP), for example, improves wetting of many synthetic membranes but may elute in amounts that vary with formulation, sterilization, storage, and rinsing [47,49,50]. For modified membranes, coating or additive stability, extractables and leachables, and retention of the intended surface properties after manufacturing, sterilization, storage, and priming should therefore be considered part of device-level characterization. Subsequent blood exposure further remodels the interface through protein deposition and secondary-membrane formation, as discussed above. The relevant unit of comparison is therefore the finished and aged dialyzer used according to its instructions, not an idealized polymer coupon [47,48,49]. Accordingly, results obtained from flat membrane samples or isolated hollow fibers should not be assigned the same evidentiary weight as findings obtained from complete dialyzers under clinically relevant flow conditions.

Treatment variables are equally important. Blood flow, ultrafiltration, hematocrit, platelet count, anticoagulant, dialysate composition, temperature, and pre-existing inflammation can change measured blood-response endpoints [4,46,51]. A membrane may also have competing effects—for example, lower cell adhesion but greater loss of a protective protein, or improved middle-molecule clearance but more albumin leakage [3,44,52]. Thus, biological effects should be interpreted together with membrane-performance variables such as permeability, solute clearance, albumin retention or loss, hydraulic stability, and, where relevant, mechanical integrity. Mechanistic interpretation should therefore use a panel of outcomes rather than a single broad “biocompatibility” or “hemocompatibility” label.

## 3. Surface-Modification Strategies and the Strength of Evidence

### 3.1. Hydrophilic and Antifouling Surfaces

Hydrophilic modification is the most mature antifouling strategy in routine HD [3,53,54]. PVP is blended into many PSf- or PES-based membranes to support pore formation and wetting; newer designs may enrich or stabilize PVP at the blood-facing surface [3,47,54]. The intended hydration layer reduces direct contact between proteins and hydrophobic polymer domains (Figure 4) [3,23]. Comparative studies report lower platelet loss, complement activation, or secondary-membrane formation for some more hydrophilic products, and a randomized trial of one modified PSf dialyzer found favorable short-term hemocompatibility markers while maintaining β2-microglobulin removal [26,47,54,55].

Polyethylene glycol (PEG) represents another hydrophilic antifouling approach. In silicon nanopore membranes developed for implantable renal-replacement systems, PEG surface modification reduced protein adsorption, fouling, and thrombosis while preserving hydraulic permeability and molecular selectivity during prolonged whole-blood hemofiltration [56]. A subsequent porcine implantable bioreactor study using PEG-coated silicon nanopore membranes also reported minimal blood-cell and protein attachment and no gross thrombus formation after implantation, although the animals received antiplatelet therapy [57]. However, these findings derive from silicon nanopore and implantable-device platforms rather than conventional disposable hollow-fiber HD, and should therefore be interpreted as mechanistic and translational support rather than direct evidence for routine hemodialysis membranes.

For currently used hydrophilic hollow-fiber dialyzers, the available data support product-specific effects on surrogate hemocompatibility outcomes rather than a class-wide reduction in clinical events. Many studies are ex vivo, involve small samples, use different comparators, or are manufacturer-sponsored [3,11,54,55]. Surface PVP must also remain sufficiently stable: excessive elution could alter surface performance or contribute to rare reactions, whereas insufficient surface coverage can weaken the hydration layer [47,58,59,60]. Reporting should include PVP content and elution after sterilization and aging, rather than describing the membrane only as “hydrophilic” [12,23,61]. Antifouling and blood-response endpoints should also be interpreted alongside permeability, solute removal, albumin loss or retention, and hydraulic stability to ensure that improved surface compatibility is not achieved at the expense of dialysis performance [3,54,55].

Zwitterionic groups such as sulfobetaine and carboxybetaine bind water strongly while maintaining near electrical neutrality [61,62]. Dialysis-oriented PES coatings have reduced fibrinogen interaction and selected complement or cellular-activation markers in vitro or ex vivo systems. The concept is persuasive, but grafting stability, scalable hollow-fiber manufacture, extractables, sterilization, and preservation of permeability remain unresolved. Accordingly, these findings are best described as antifouling or endpoint-specific hemocompatibility improvements rather than established anti-inflammatory efficacy. No patient-centered benefit can yet be inferred from these laboratory results.

### 3.2. Antithrombotic and Complement-Directed Interfaces

Immobilizing an anticoagulant at the blood-facing surface seeks to suppress local thrombin generation without requiring equivalent systemic anticoagulant exposure (Figure 5) [63,64,65]. The best clinical example is a heparin-grafted dialyzer [66,67]. In the multicenter HepZero trial of patients at increased bleeding risk, the heparin-grafted membrane achieved a higher proportion of successful heparin-free treatments than the center’s standard heparin-avoidance strategy [66]. The composite endpoint considered treatment unsuccessful when clinically relevant clotting led to complete occlusion, additional saline flushing, replacement of the dialyzer or blood lines, or premature termination of the session. Despite the advantage of the heparin-grafted membrane, treatment success was not universal [66]. This device can facilitate heparin-free HD in selected patients, but it does not abolish clotting or replace clinical surveillance [66,68].

Other anticoagulant surface strategies remain largely preclinical. These include heparin and heparin-mimetic coatings, hirudin-based surfaces, and immobilized direct thrombin inhibitors [63,64,65,69]. A recent heparin-immobilized PES membrane showed reduced fibrinogen adsorption together with improved ex vivo blood-contact markers [69]. Translation requires evidence that the molecule remains surface-bound, retains activity after sterilization, does not create clinically meaningful systemic exposure, and continues to provide antithrombotic activity without compromising membrane transport performance during treatment [64,65,69]. Anticoagulant surfaces should also be tested with low, clinically relevant background anticoagulation rather than only in static plasma clotting assays [68,70].

Complement-directed coatings, including immobilized regulators or complement-binding structures, are an appealing extension of this approach (Figure 5) [71,72]. The aim is to limit complement activation at the blood-contacting surface and thereby reduce downstream generation of inflammatory complement fragments and leukocyte recruitment. However, complement is also important for antimicrobial defense, and its surface regulation must be spatially restricted and durable [73,74]. Most evidence for factor H-binding structures and soluble complement receptor 1-based coatings still comes from experimental biomaterial systems. Direct evidence in dialysis membranes remains limited, and improvement in clinical outcomes has not been established [6,72,75]. At present, these are research targets, not established components of HD membranes.

### 3.3. Antioxidant Functionalization

Vitamin E-bonded membranes are the most extensively studied antioxidant modification [76,77]. By locating a lipid-soluble antioxidant at the blood-facing surface, these membranes aim to limit lipid and protein oxidation during repeated treatments (Figure 6) [78,79]. Meta-analyses have reported improvements in some surrogate measures related to oxidative stress, inflammation, and anemia, but the included trials were generally small and heterogeneous in membrane composition, comparator, duration, and assay [76,80,81]. These findings support an effect on selected biochemical and clinical laboratory endpoints, but they should not be interpreted as evidence of a general anti-inflammatory benefit. The evidence does not establish a reduction in cardiovascular events, hospitalization, or mortality.

Natural antioxidants such as resveratrol have been incorporated into PSf membranes and have shown radical-scavenging activity and improved blood-contact measures in laboratory studies [82]. Such experiments are useful demonstrations of feasibility, not clinical efficacy. Covalent attachment may reduce release but can change molecular activity; blending can increase exposure to leachables. The durability of antioxidant activity after sterilization and blood exposure is therefore as important as the initial antioxidant capacity. Antioxidant capacity should therefore be measured after sterilization and simulated use, alongside cytotoxicity, solute transport, permeability, albumin retention or loss, hydraulic performance, and chemical characterization [48,77,82]. An antioxidant surface should only be considered advantageous when the reduction in oxidative stress is achieved without compromising dialysis performance.

### 3.4. Intrinsic Material Design and Adsorptive Surfaces

Not every reduction in inflammatory markers is due to a surface modification [43,83,84]. Changes in membrane composition, pore architecture, and intrinsic adsorption behavior may alter blood-contact responses without involving a deliberately engineered surface coating. Cellulose triacetate, PSf/PES, polyacrylonitrile-derived membranes, and polymethylmethacrylate differ in bulk chemistry, pore structure, and adsorption [2,18,85,86,87]. Proteomic studies show that asymmetric cellulose triacetate, conventional cellulose triacetate, and synthetic membranes acquire distinct adsorbed-protein profiles [18,84,88]. Adsorption may be useful when a target mediator is removed, but it can also deplete beneficial proteins or mask whether lower plasma concentrations reflect reduced production or greater capture [43,83,89,90]. Accordingly, a lower circulating concentration of a cytokine or complement-related protein should not automatically be interpreted as reduced inflammatory activation.

Material and surface approaches should therefore be compared using a multidimensional profile: small- and middle-molecule removal, albumin loss, hydraulic stability, protein adsorption, complement, platelets, coagulation, leukocytes, hemolysis, extractables, and clinical tolerability [48,91,92,93]. These dimensions should be considered together because improvement in one endpoint may coexist with deterioration in another. A single polymer name, adsorption measurement, or cytokine concentration therefore cannot define the overall hemocompatibility or clinical value of a dialyzer.

## 4. Translational Evaluation of Modified Dialyzers

### 4.1. In Vitro and Ex Vivo Hemocompatibility

International Organization for Standardization (ISO) 10993-4 provides the general framework for evaluating interactions between medical devices and blood, including thrombosis, coagulation, platelet, hematological, and complement-related endpoints [94,95,96,97]. For a dialyzer, one of the most informative early models is a dynamic loop or recirculation system containing fresh human whole blood, a clinically representative surface area, controlled temperature, blood and dialysate flow, and a justified anticoagulant concentration [46,51,98,99,100]. Such dynamic systems are particularly useful for device-level testing because they better reproduce flow-dependent blood–surface interactions than static material assays. Static incubation is useful for screening chemistry but does not reproduce shear, concentration polarization, filtration, or the evolving secondary membrane [95,96,99,101].

A robust panel should include platelet count and activation, thrombin generation or thrombin–antithrombin complexes, complement fragments, leukocyte activation, hemolysis, and direct imaging or quantification of surface deposition [95,98,102]. Activated partial thromboplastin time (APTT) and prothrombin time (PT) mainly describe plasma clotting under assay conditions and are poor stand-alone surrogates for circuit thrombosis [65,99,103,104]. Assay selection should therefore be aligned with the specific biological effect being claimed, rather than using an isolated coagulation, complement, platelet, or hemolysis endpoint to support a broad claim of overall hemocompatibility. Recent full-dialyzer work illustrates how platelet, coagulation, and complement endpoints can be combined in a human-blood recirculation model within the broader framework recommended for blood-contacting devices [98]. However, ISO 10993-4 provides general principles for test selection rather than a dialyzer-specific experimental protocol, so use of an individual recirculation model should not by itself be interpreted as proof of full compliance with the standard. Because dialysate and electrolyte composition can affect results, the complete test configuration must be reported [51,98].

Controls are essential: a loop without the test device, a marketed comparator with similar surface area and performance, and, when scientifically justified, a more activating positive control [96,99,100]. Samples should be collected at baseline and at several early and late time points because pathways peak at different times [51,96,98,105]. Donor number, exclusion criteria, time from phlebotomy, collection system, anticoagulant, and statistical handling of repeated measures should be prespecified. The exact dialyzer, sterilization method, priming procedure, tubing, surface area, and flow conditions should also be documented when complete devices are tested. Claims of “excellent hemocompatibility” based on one donor, one marker, or an uncorrected multiple comparison are not persuasive. When complete dialyzers are compared, hemocompatibility findings should also be considered together with clearance, albumin retention or loss, and hydraulic performance.

### 4.2. Animal Models: A Selective Rather than Routine Bridge

Animal studies used in the evaluation of blood-contacting dialysis materials include extracorporeal models that reproduce blood circulation through a functioning device and implantation models that primarily assess local material–tissue responses. These approaches address different questions and should not be assigned the same translational relevance when evaluating dialyzer hemocompatibility. Implantation-site inflammation in a rodent does not model a disposable hollow-fiber dialyzer, which contacts flowing blood transiently and is not intended to endothelialize or integrate with tissue [95,96,99,106]. Implantation tests may address a separate material hazard, but they cannot establish dialyzer hemocompatibility [96,107]. They are therefore more appropriate for questions related to local tissue response or material safety than for predicting the acute blood response to a functioning dialyzer. Species differences in complement, platelets, coagulation, and responses to anticoagulants also limit direct translation [96,99,108,109]. Red blood cell susceptibility to mechanical injury is also species-dependent, which is relevant when hemolysis is used as a safety endpoint. Under standardized shear conditions, ovine blood has shown greater susceptibility to hemolysis than bovine, porcine, and human blood, while bovine and porcine responses were closer to those of human blood [110]. For this reason, favorable findings in a rodent implantation model should not be presented as direct evidence that a membrane will show improved hemocompatibility during human HD.

Large-animal extracorporeal circuits can answer focused questions that cannot be resolved in vitro. A porcine model has reproduced acute complement and cardiopulmonary responses to dialyzer exposure, and a short-term nephrectomized-pig model has been developed for repeated HD [111,112]. These extracorporeal approaches are more relevant than subcutaneous implantation but remain resource-intensive and may not reproduce the chronic immune and vascular phenotype of human kidney failure [96,111,112]. Their main value lies in addressing defined system-level questions, such as acute cardiopulmonary responses, whole-circuit effects, or repeated extracorporeal exposure that cannot be adequately reproduced in human-blood loop models. Animal models should therefore be used selectively rather than as a routine requirement for every modified membrane. Within a staged translational pathway, dynamic human-blood testing should usually precede animal studies. Animal experiments are most informative when they address a specific unresolved safety or physiological question and when the selected species and experimental configuration are justified for that purpose. Even then, animal findings should be interpreted as supportive evidence rather than a substitute for controlled human studies.

### 4.3. From Biomarkers to Clinical Evidence

Early clinical studies should use randomized within-patient crossover designs when feasible, identical anticoagulation and treatment prescriptions, adequate washout or period modeling, and sampling that captures early complement peaks as well as end-of-session responses [44,54,55,113,114]. Device performance and safety outcomes—clearance, albumin loss, transmembrane pressure, platelet loss, circuit clotting, treatment interruption, and hypersensitivity—should be reported alongside molecular biomarkers [54,55,66,68,115]. Acute changes in complement fragments, platelet activation, cytokines, or oxidative-stress markers should be interpreted primarily as mechanistic or surrogate evidence unless they are prospectively linked to clinically relevant outcomes. A favorable biomarker profile is more informative when it occurs together with preserved solute removal, acceptable albumin loss, and stable circuit performance.

Later trials need endpoints that matter to patients. Potential outcomes include successful treatment without excess anticoagulation, clinically significant clotting, transfusion or bleeding, dialysis reactions, infection, hospitalization, symptoms, quality of life, cardiovascular events, and survival [66,68,116,117]. Large trials comparing membrane flux or permeability show why mechanistic plausibility and better clearance do not automatically translate into mortality benefit [117,118,119]. Likewise, systematic reviews of medium cut-off versus high-flux membranes found clearer advantages in biomarker and middle-molecule removal than in major clinical outcomes, with definitive evidence for mortality, hospitalization, and serious adverse events remaining limited [44,116,120]. The evidence should therefore be described in levels, with biomarker responses separated from treatment-level outcomes and from longer-term patient-centered benefit. Improvement at one level should not be assumed to establish benefit at the next. Manufacturing evidence is part of clinical translation [48]. The final device should be characterized after sterilization and shelf aging for surface composition, coating integrity, extractables and leachables, hydraulic and mechanical performance, and batch-to-batch variability [47,50,121]. This is particularly important for surface-modified membranes because the chemistry evaluated in a fresh laboratory sample may not fully represent the blood-facing interface of the commercial device after manufacturing, storage, and priming. Studies detecting bisphenol A or bisphenol S from dialyzer components illustrate that exposure can arise from the complete device rather than the membrane surface alone [48,122]. A modification that improves one activation marker but introduces unstable chemistry, clinically relevant leaching, or impaired dialysis performance should therefore not be considered a net clinical advance. A comparative summary of the major membrane-modification strategies, their hemocompatibility targets, effects on membrane performance, and translational evidence is provided in Table 1.

## 5. Future Directions

The next useful membrane is more likely to emerge from disciplined integration than from adding the largest number of bioactive components. Hydration, local antithrombotic activity, controlled adsorption, and antioxidant function could be combined, but each added function increases analytical, manufacturing, toxicological, and regulatory burden [53]. Multifunctional design should therefore follow, rather than replace, validation of each individual component and should preserve solute transport, albumin retention, hydraulic stability, and chemical durability. Development should begin with a prespecified clinical problem—for example, recurrent platelet loss or inability to use systemic anticoagulation—and add only mechanisms that measurably address it [123,124].

Omics and computational tools can help map the interface. Proteomics can identify adsorbed networks rather than isolated proteins, while molecular modeling and machine learning may prioritize surface chemistries [17]. A recent machine-learning model predicted protein–membrane interaction energies from a molecular-docking dataset [125]. Such models are hypothesis generators: performance against the training distribution does not establish prediction of a dynamic protein corona, whole-blood activation, or patient outcomes. Independent experimental validation is essential before computational predictions are used to guide claims of improved hemocompatibility. Models should ultimately be tested against dynamic blood-contact systems and complete dialyzers rather than only against the datasets from which they were developed.

Responsive drug release and closed-loop sensing remain preclinical or early-stage concepts for intermittent HD. A trigger must vary reliably in circulating blood, distinguish harmful device activation from baseline uremia, and operate within minutes without releasing an unsafe systemic dose. Tissue-acidosis paradigms cannot simply be transferred to an extracorporeal blood compartment. Similarly, immobilized cytokine antibodies, Toll-like-receptor antagonists, nitric-oxide donors, or complement regulators should not be presented as validated dialysis-membrane therapies until binding stability, dose, sterility, leachables, and dialysis-specific efficacy have been demonstrated. These approaches are better regarded as exploratory bioactive-device strategies until their advantages over simpler surface modifications are demonstrated under clinically relevant dialysis conditions.

Progress would be accelerated by a core outcome set for dialyzer hemocompatibility, reference devices, open protocols for dynamic blood testing, and independent replication. Clinical studies should report the exact product and sterilization method, register protocols, disclose manufacturer involvement, and link acute biomarkers to prespecified clinical outcomes. Future studies should also state clearly whether evidence is derived from flat materials, hollow fibers, complete dialyzers, dynamic blood-circulation systems, or patients, because these models provide different levels of translational evidence. Patient stratification may also matter: a surface modification that is marginal in an unselected population could be valuable in patients with recurrent dialyzer reactions, thrombocytopenia, or high bleeding risk. Such targeted use cases may provide a more realistic route to clinical benefit than seeking one universally “anti-inflammatory” membrane for all patients.

## 6. Discussion

The available evidence supports an interconnected biological network at the blood–membrane interface: rapid protein adsorption creates a secondary surface; complement and contact pathways are activated to varying degrees; and platelets, neutrophils, and monocytes respond through adhesive, procoagulant, and oxidative mechanisms [4,126]. These events overlap and reinforce one another rather than occurring as a strictly linear cascade. This network is more than a laboratory artifact, because relevant markers change during clinical HD and surface-specific differences are reproducible in several controlled systems. The uncertainty lies not in whether interactions occur, but in how much they contribute to chronic inflammation and patient outcomes in the setting of multiple competing causes.

That distinction changes how membrane technologies should be described. Hydrophilic surfaces have the most consistent evidence for reducing fouling and selected activation markers, but results remain product-specific [3,10,55]. A heparin-grafted dialyzer has clinical evidence for facilitating heparin-free treatment in a selected high-bleeding-risk population, yet incomplete circuit success prevents it from being described as a universal replacement for anticoagulation [66,68]. Vitamin E-coated membranes have shown improvements in selected surrogate outcomes, particularly markers related to oxidative stress, inflammation, and anemia, whereas cardiovascular and survival benefits remain unproven. Zwitterionic surfaces, other experimental immobilized-heparin approaches, and resveratrol-modified membranes are technically promising but should still be labeled preclinical.

Several concepts in the broader biomaterials literature are poorly matched to a disposable dialyzer. Endothelialization and growth-factor-driven tissue integration are relevant to long-term implants, not to a membrane discarded after a treatment. Likewise, placing cytokine-neutralizing antibodies or a stimulus-responsive drug reservoir in direct contact with blood converts a relatively passive device into a complex bioactive delivery system [53]. Such approaches may eventually be feasible, but they require a dialysis-specific pharmacological rationale and safety program; extrapolation from vascular grafts, wound materials, or inflamed tissue is not sufficient.

A further limitation of the field is the interchangeable use of “biocompatibility,” “hemocompatibility,” and “anti-inflammatory.” A membrane can show a lower C3a level but similar platelet activation, or lower cell adhesion but greater protein loss [3,4,10,54]. These are different dimensions, not contradictory findings. Claims should identify the endpoint, model, comparator, and time point. This discipline would also reduce the risk that statistically significant changes in a single biomarker are presented as clinically meaningful improvements.

The current review has limitations. It is narrative rather than systematic, the underlying studies use heterogeneous membranes and assays, and many modern product studies are small or involve manufacturers. Publication bias is likely, particularly for novel coatings. In addition, long-term trials rarely isolate surface chemistry from flux, pore distribution, treatment modality, anticoagulation, and device geometry. Accordingly, this review can rank the maturity of evidence but cannot identify one membrane as optimal for every patient.

The most convincing evidence will therefore come from a staged translational framework that links stable surface chemistry and preserved dialysis performance with reproducible dynamic blood testing and, where appropriate, targeted animal and clinical studies. Mechanistic biomarkers should ultimately be connected to circuit performance and patient-centered outcomes before a surface is described as clinically anti-inflammatory.

## 7. Conclusions

Blood–membrane interactions in HD are driven by a linked protein, complement, coagulation, platelet, and leukocyte network rather than by a single pathway. Surface hydrophilization, heparin grafting, and vitamin E coating have reached clinical use, but their evidence and indications differ, and none has established a general reduction in long-term cardiovascular risk. Zwitterionic surfaces, experimental immobilized-anticoagulant coatings, and antioxidant small-molecule modifications such as resveratrol remain predominantly preclinical. Improvement in acute hemocompatibility markers should therefore not be interpreted as established clinical benefit without corresponding evidence from treatment-level and patient-centered outcomes. Future dialyzers should be judged as complete devices using standardized dynamic blood testing, chemical and manufacturing characterization, preserved clearance, and patient-centered clinical endpoints. Cautious, endpoint-specific claims will make advances in membrane science more credible and more clinically useful.

## Figures and Tables

**Figure 1:**
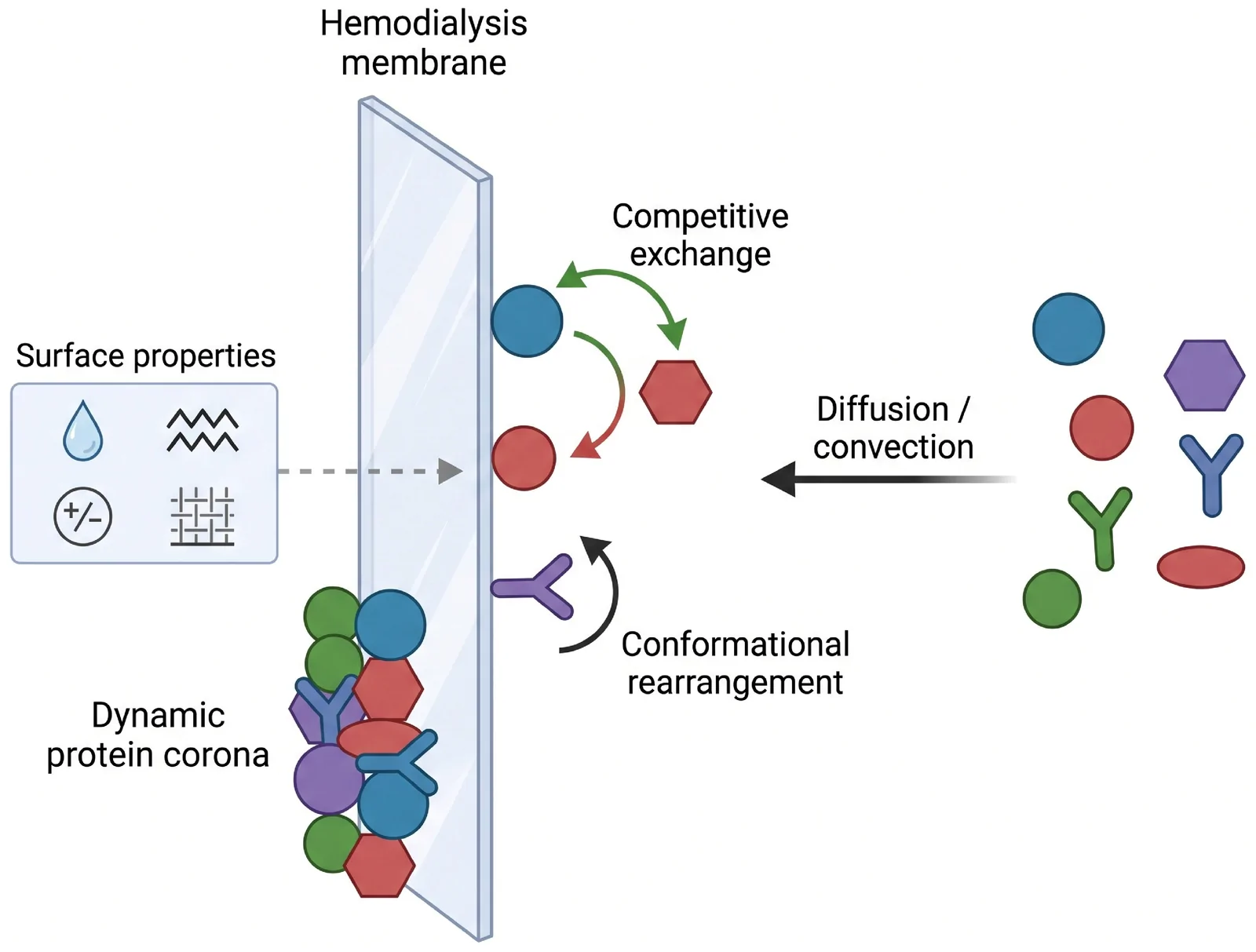
Dynamic formation of the protein corona at the hemodialysis membrane. Plasma proteins reach the blood-facing membrane surface through diffusion and convection and undergo competitive adsorption and exchange. Adsorbed proteins may subsequently undergo conformational and orientational rearrangements. Membrane surface properties, including hydration, hydrophobicity, surface charge, and nanoscale roughness, modulate these processes, ultimately generating a dynamic protein corona at the blood–membrane interface. Colors are used only to visually distinguish representative proteins and do not denote specific protein classes.

**Figure 2:**
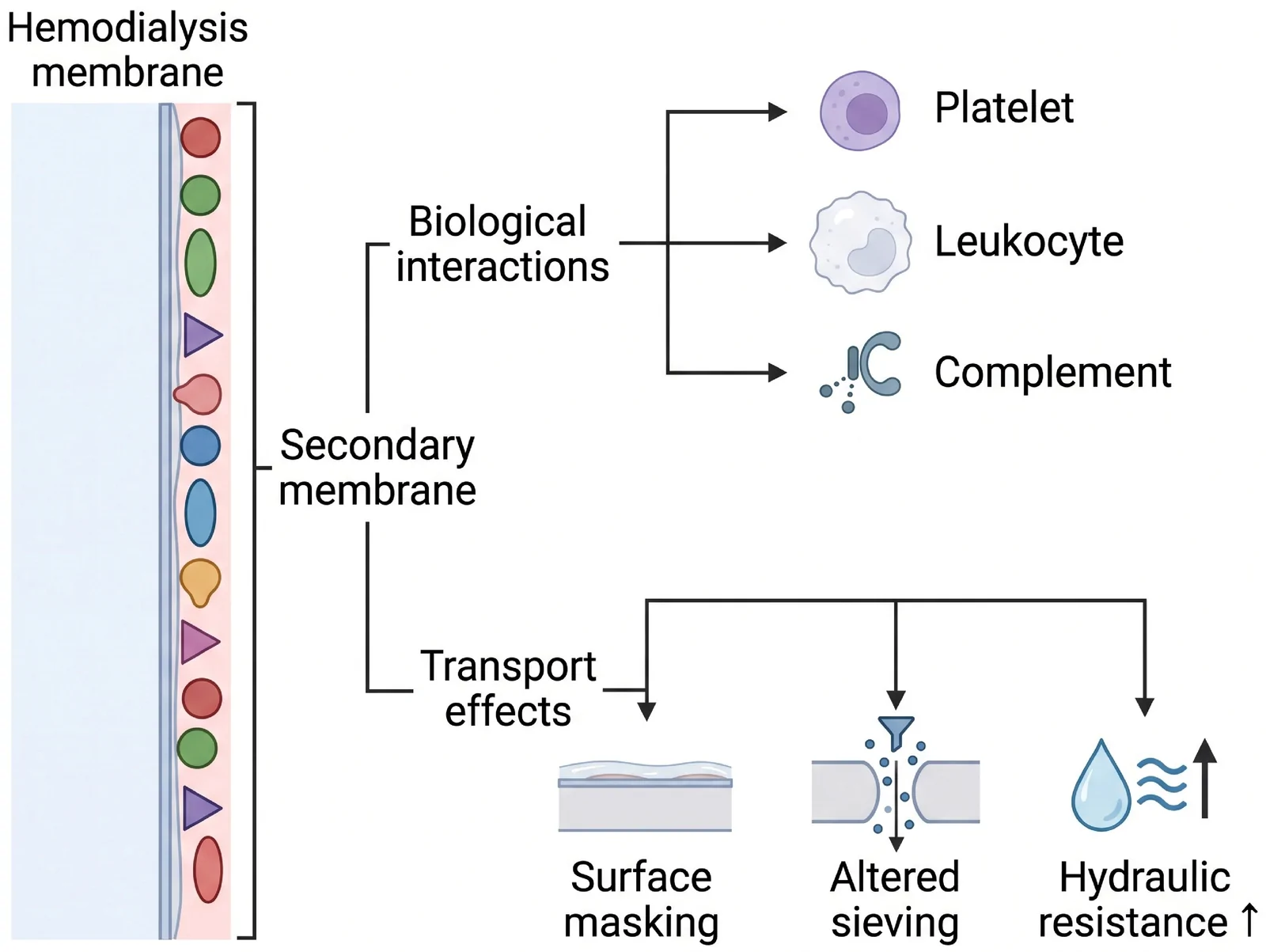
Functional consequences of secondary-membrane formation during hemodialysis. The adsorbed-protein layer functions as a secondary membrane that can provide binding interfaces for platelets, leukocytes, and complement. In parallel, the secondary membrane can mask the original membrane surface and alter transport properties, including solute sieving and hydraulic resistance. These biological and transport effects illustrate why protein adsorption should be evaluated as both a hemocompatibility and membrane-performance phenomenon.

**Figure 3:**
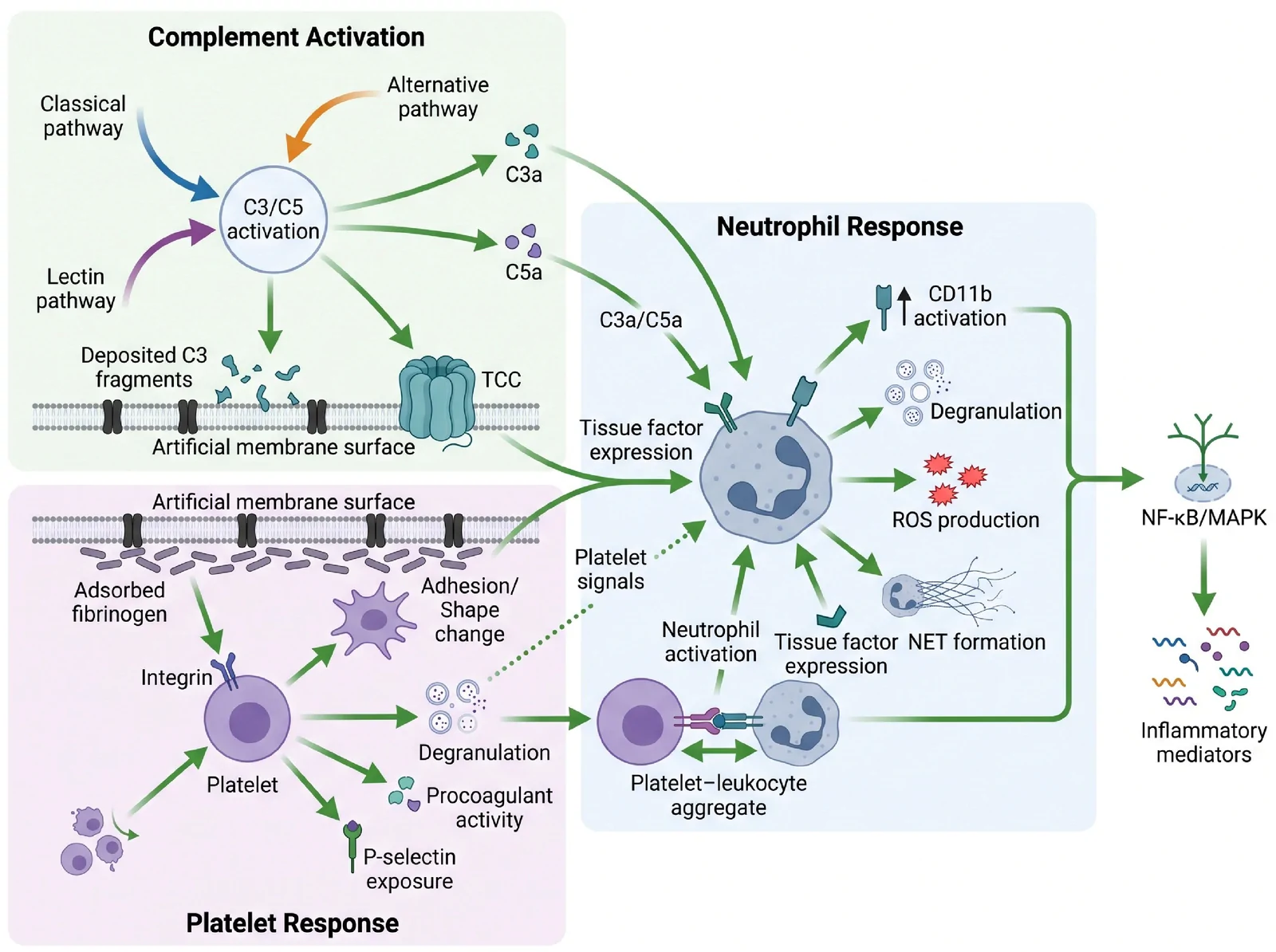
Interconnected complement, platelet, and neutrophil responses during hemodialysis. Alternative, lectin, and classical complement pathways converge at C3 and C5 to generate C3a, C5a, deposited C3 fragments, and terminal complement complexes. Adsorbed fibrinogen promotes platelet integrin engagement, adhesion, degranulation, procoagulant activity, and P-selectin exposure. Complement fragments, platelet signals, and the adsorbed surface contribute to neutrophil CD11b activation, degranulation, reactive oxygen species production, tissue-factor expression, neutrophil extracellular trap formation, and platelet–leukocyte aggregation. These interconnected responses engage NF-κB- and MAPK-related signaling and inflammatory mediator release. Abbreviations: TCC, terminal complement complex; ROS, reactive oxygen species; NET, neutrophil extracellular trap; NF-κB, nuclear factor kappa B; MAPK, mitogen-activated protein kinase. Arrows indicate the direction of activation or interaction, while colors are used to visually distinguish pathways and cellular components and do not indicate quantitative differences.

**Figure 4:**
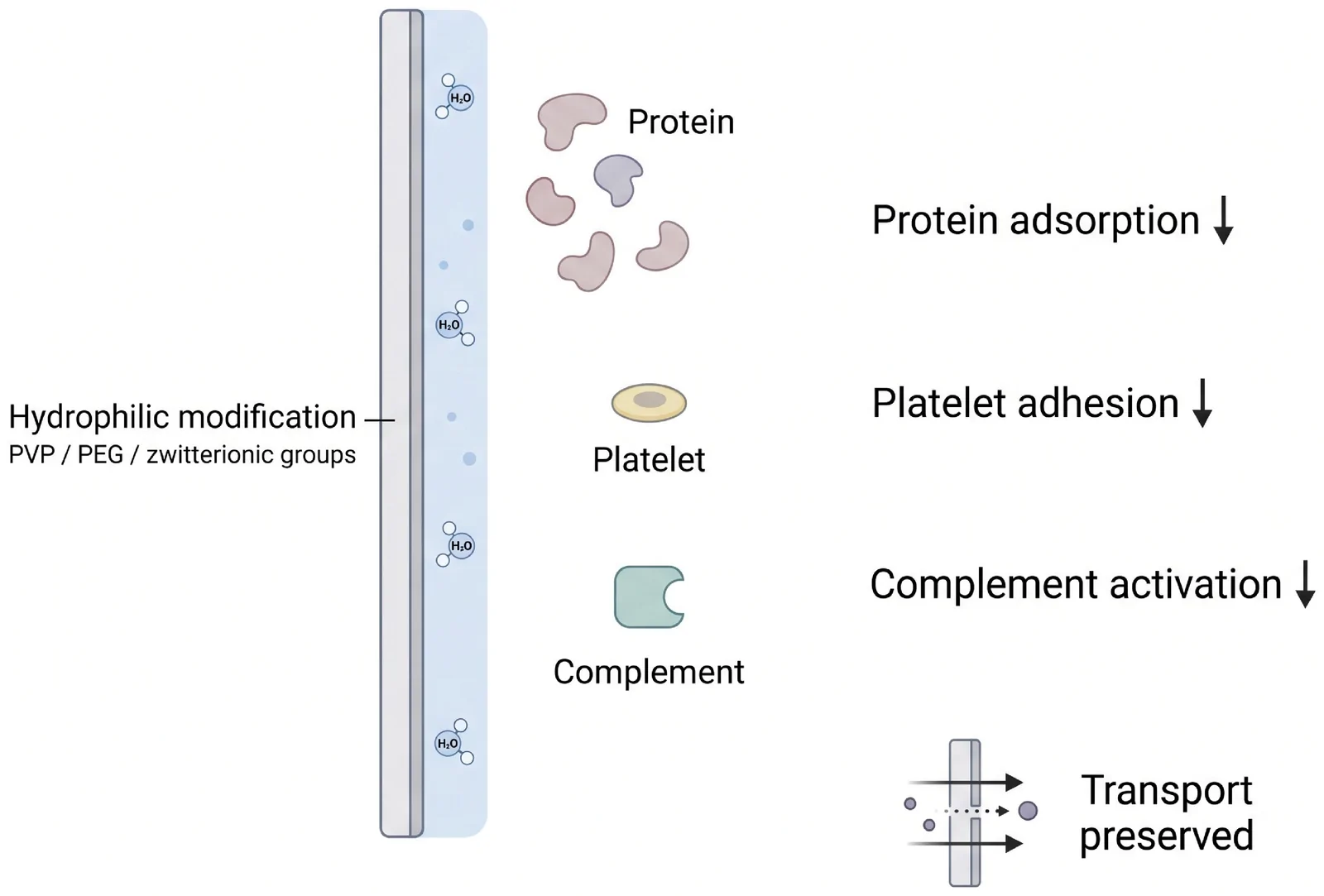
Hydrophilic and antifouling modification of the hemodialysis membrane. Representative strategies, including polyvinylpyrrolidone (PVP), polyethylene glycol (PEG), and zwitterionic groups, promote formation of a hydrated blood-facing interface that reduces direct protein–polymer contact. These surface modifications are associated with reduced protein adsorption, platelet adhesion, and complement activation while aiming to preserve membrane transport performance. Downward arrows indicate reductions in protein adsorption, platelet adhesion, and complement activation, while the horizontal arrows indicate preserved membrane transport.

**Figure 5:**
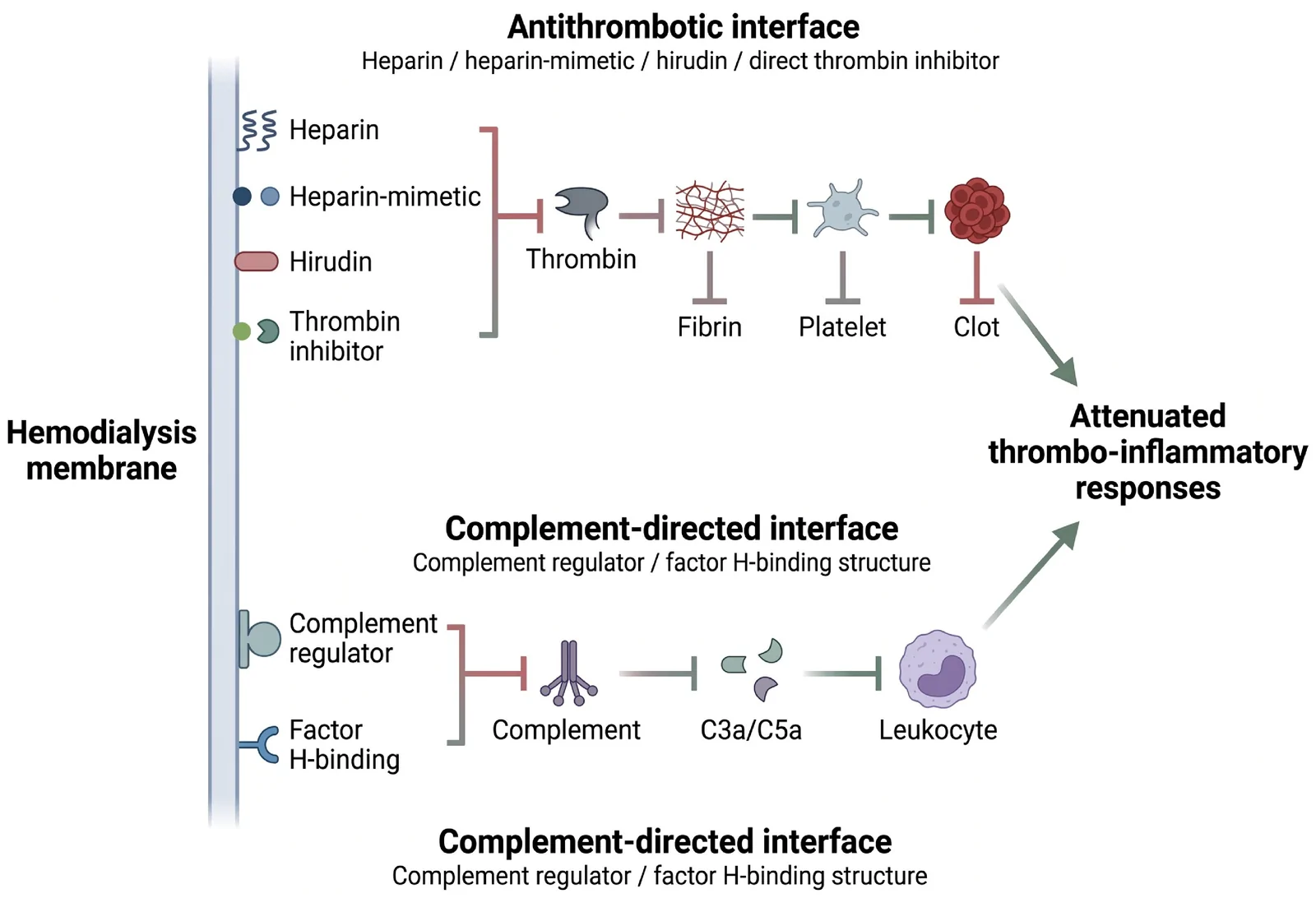
Antithrombotic and complement-directed surface strategies for hemodialysis membranes. Surface-bound anticoagulant approaches, including heparin, heparin mimetics, hirudin, and direct thrombin inhibitors, are intended to reduce local thrombin generation, fibrin formation, platelet activation, and clot formation. Complement-directed interfaces, including immobilized complement regulators and factor H-binding structures, aim to attenuate surface complement activation, C3a/C5a generation, and leukocyte recruitment. Together, these approaches are designed to limit thrombo-inflammatory responses at the blood–membrane interface. Arrows indicate the direction of the proposed interactions, while inhibitory bars indicate suppression of thrombin- and complement-related processes. Colors are used for visual distinction only.

**Figure 6:**
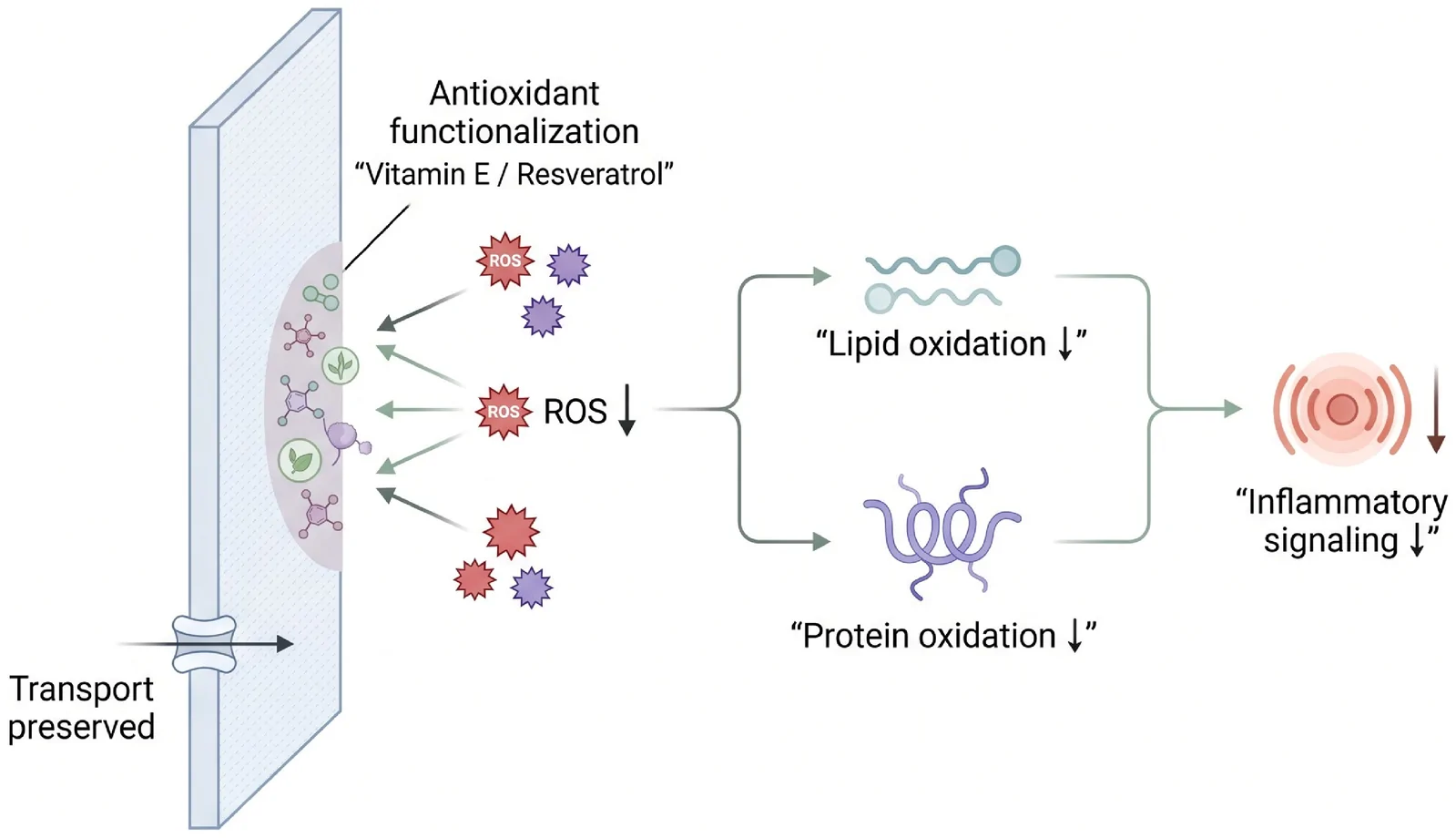
Antioxidant functionalization of hemodialysis membranes. Surface-associated vitamin E or resveratrol is intended to reduce reactive oxygen species at the blood–membrane interface, thereby limiting lipid and protein oxidation and downstream inflammatory signaling while preserving membrane transport function. Abbreviation: ROS, reactive oxygen species. Downward arrows indicate reductions in ROS, lipid oxidation, protein oxidation, and inflammatory signaling. Colors are used for visual distinction only.

**Table 1:** Comparative summary of hemodialysis membrane modification strategies, hemocompatibility targets, membrane-performance considerations, and translational evidence.

Strategy/Representative Material	Modification Method	Proposed Mechanism/Primary Target	Main Findings	Experimental Model/Evidence Level	Membrane-Performance Considerations	Key Limitations	References
PVP-based hydrophilic membranes	PVP blended into PSf/PES or enriched at the blood-facing surface	Formation of a hydrated interface that reduces direct protein–polymer contact and surface fouling	Some products show reduced platelet loss, complement activation, or secondary-membrane formation. Randomized studies have reported favorable short-term hemocompatibility markers	Complete dialyzers, ex vivo comparisons, randomized clinical studies. Clinical use with mainly surrogate, product-specific evidence	β2-microglobulin removal can be maintained, but permeability, albumin loss, and hydraulic stability should be assessed together with hemocompatibility	Product-specific effects, PVP elution, incomplete surface coverage, sterilization and aging effects	[3,47,54,55,58,59,60]
PEG-modified surfaces	PEG surface coating	Hydrophilic steric barrier that limits protein adsorption, fouling, and cellular attachment	PEG modification reduced protein adsorption, fouling, and thrombosis in silicon nanopore membranes during prolonged whole-blood hemofiltration	Silicon nanopore membranes, prolonged whole-blood hemofiltration, and a porcine implantable bioreactor. Preclinical/translational evidence	Hydraulic permeability and molecular selectivity were preserved in the reported platform	Evidence derives from an implantable silicon nanopore system rather than conventional disposable hollow-fiber HD	[56,57]
Zwitterionic PES surfaces	Immobilization or grafting of sulfobetaine, carboxybetaine, or related zwitterionic groups	Strong surface hydration with near-neutral net charge, reducing protein interaction and cell activation	Reduced fibrinogen interaction and selected complement or cellular-activation markers have been reported	Flat membranes, modified PES membranes, in vitro and ex vivo systems. Preclinical evidence	Permeability and pore characteristics must be preserved after grafting	Grafting stability, scalable hollow-fiber manufacture, extractables, sterilization, and limited clinical validation	[12,23,61,62]
Heparin-grafted clinical dialyzers	Covalent or stable surface grafting of heparin	Local reduction in thrombin generation and circuit thrombogenicity with less systemic anticoagulation	HepZero and related studies support heparin-free or reduced-anticoagulation HD in selected patients, although clotting is not abolished	Complete dialyzers and prospective clinical trials. Clinical evidence for selected indications	Bulk dialysis performance should be preserved, while circuit patency and treatment completion remain key device-level outcomes	Treatment success is not universal and clinical surveillance remains necessary	[66,67,68,70]
Experimental heparin, heparin-mimetic, hirudin, and direct thrombin inhibitor surfaces	Immobilization of anticoagulant molecules on the blood-facing surface	Local inhibition of thrombin, fibrin formation, platelet activation, or related coagulation processes	Experimental heparin-modified PES has shown reduced fibrinogen adsorption and improved ex vivo blood-contact markers	Material-level and ex vivo blood-contact models. Predominantly preclinical evidence	Antithrombotic activity should be maintained without reducing permeability, solute transport, or hydraulic performance	Surface retention, activity after sterilization, possible systemic exposure, durability, and limited clinical testing	[63,64,65,69]
Complement-directed surfaces	Immobilized complement regulators or factor H-binding structures	Local attenuation of complement activation, C3a/C5a generation, and downstream leukocyte recruitment	Experimental coatings can reduce complement-related activation in biomaterial systems	Experimental biomaterial systems with limited dialysis-specific testing. Preclinical evidence	Effects on permeability and complete-dialyzer performance remain insufficiently characterized	Durability, spatial restriction of complement inhibition, antimicrobial-defense concerns, and lack of clinical dialysis evidence	[6,71,72,73,74,75]
Vitamin E-coated membranes	Surface association or bonding of vitamin E	Antioxidant activity intended to limit lipid and protein oxidation during blood contact	Meta-analyses report improvements in selected oxidative-stress, inflammatory, and anemia-related surrogate outcomes	Clinical studies and meta-analyses. Clinical use with mainly surrogate-outcome evidence	Antioxidant benefit should occur without compromising permeability, clearance, albumin handling, or hydraulic performance	Heterogeneous trials, variable comparators and assays, and no established reduction in cardiovascular events, hospitalization, or mortality	[76,77,78,79,80,81]
Resveratrol-functionalized PSf membranes	Incorporation or immobilization of resveratrol	Radical scavenging and reduction in oxidative blood–material interactions	Laboratory studies show antioxidant activity and improved blood-contact measures	Modified PSf membranes and laboratory studies. Preclinical evidence	Permeability, albumin retention, hydraulic performance, and chemical stability require evaluation after modification	Stability, leaching, sterilization, maintenance of molecular activity, and absence of clinical efficacy data	[82]
Intrinsic and adsorptive membrane designs	Modification of bulk polymer chemistry, pore architecture, or intrinsic adsorption rather than addition of a discrete surface coating	Selective adsorption and material-dependent modulation of protein and cellular interactions	CTA, asymmetric CTA, PSf/PES, polyacrylonitrile-derived, and PMMA membranes show different adsorbed-protein profiles and blood-contact responses	Proteomic, material-comparison, and complete-dialyzer studies. Heterogeneous, product-specific evidence	Adsorption can affect solute removal, albumin loss, secondary-membrane formation, and hydraulic behavior	Lower circulating mediator concentrations may reflect adsorption rather than reduced biological production. Polymer name alone does not define hemocompatibility	[2,18,83,84,85,86,87,88,89,90,91,92,93]

Abbreviations: PVP, polyvinylpyrrolidone; PEG, polyethylene glycol; PSf, polysulfone; PES, polyethersulfone; HD, hemodialysis; CTA, cellulose triacetate; PMMA, polymethylmethacrylate.

## Data Availability

No new data were created or analyzed in this study. Data sharing is not applicable to this article.
